# Initiating and continuing participation in citizen science for natural history

**DOI:** 10.1186/s12898-016-0062-3

**Published:** 2016-07-22

**Authors:** Glyn Everett, Hilary Geoghegan

**Affiliations:** 1 Faculty of Environment and Technology, University of the West of England, Frenchay Campus, Coldharbour Lane, Bristol, BS16 1QY UK; 2 Department of Geography and Environmental Science, University of Reading, Whiteknights, Reading, RG6 6DW UK

## Abstract

**Background:**

Natural history has a long tradition in the UK, dating back to before Charles Darwin. Developing from a principally amateur pursuit, natural history continues to attract both amateur and professional involvement. Within the context of citizen science and public engagement, we examine the motivations behind citizen participation in the national survey activities of the Open Air Laboratories (OPAL) programme, looking at: people’s experiences of the surveys as ‘project-based leisure’; their motivations for taking part and barriers to continued participation; where they feature on our continuum of engagement; and whether participation in an OPAL survey facilitated their movement between categories along this continuum. The paper focuses on a less-expected but very significant outcome regarding the participation of already-engaged amateur naturalists in citizen science.

**Results:**

Our main findings relate to: first, how committed amateur naturalists (already-engaged) have also enjoyed contributing to OPAL and the need to respect and work with their interest to encourage broader and deeper involvement; and second, how new (previously-unengaged) and relatively new participants (casually-engaged) have gained confidence, renewed their interests, refocussed their activities and/or gained validation from participation in OPAL. Overall, we argue that engagement with and enthusiasm for the scientific process is a motivation shared by citizens who, prior to participating in the OPAL surveys, were previously-unengaged, casually-engaged or already-engaged in natural history activities.

**Conclusions:**

Citizen science has largely been written about by professional scientists for professional scientists interested in developing a project of their own. This study offers a qualitative example of how citizen science can be meaningful to participants beyond what might appear to be a public engagement data collection exercise.

## Background

Citizen science is defined here as the participation of non-professional scientists in observation and recording for professional science projects [1]. Citizen scientists have been heralded as one solution to a crisis of monitoring and shortage of data in the field [2–6]. Historically, networks of natural historians have made essential contributions to the acquisition of taxonomic data [7]. Notwithstanding other monitoring activities, the Audubon Christmas Bird Count is widely regarded as the first ‘citizen science’ exercise in the field of natural history, starting in 1900 and continuing through to the present day [8, 9].

Since the mid-1930s, volunteer naturalists– rather than professional taxonomists—have formed an ‘army of new recorders’ [10] recruited by initiatives such as the British Trust for Ornithology’s Nest Record Scheme and the Royal Society for the Protection of Birds’ Big Garden Birdwatch. With millions of people contributing to such schemes on an annual basis [2], a recent report regarding the state of UK taxonomy stated that: ‘The voluntary sector, with its core of expert amateur naturalists, is an important repository of taxonomic expertise. The volunteers monitor changes in their local fauna and flora, provide records for biological recording schemes, and generate data for Biodiversity Action Plans’ [7].

Today there is a concern (in the UK and the US at least) that we are seeing a ‘decline in numbers of both amateur and professional taxonomists’ [11] and that volunteer efforts in the area of biodiversity recording have been subject to a general decline in numbers. It has been suggested, in a study conducted for the House of Lords in the UK and elsewhere, that the relative strength of the amateur naturalist community as a ‘workforce’ of taxonomy [11] is fading and that the ability to recruit and train new generations of naturalists is a struggle [12, 13]. Indeed, much has been written about the decline, death or ‘impending extinction’ of natural history as both an academic subject and amateur enthusiasm [14–18]. For Anna Lawrence [19], ‘specialist amateurs are on the decline while more generalist volunteers and environmental enthusiasts are on the rise’.

Notwithstanding professionals working in this area, it appears that our fascination with natural history has shifted from one of keen amateurism to a casual leisure interest with fewer people actively recording and contributing data. This is a concern for many, who argue that there is a ‘dearth of basic knowledge’ just as our need for knowledge is increasing due to the loss of biodiversity [20, 21]; many biologists today refer to the past 500 years as that of a sixth mass (and first grand anthropogenic) extinction [22–25]. Central to any understanding of and response to changes in flora and fauna is the participation of an adequately trained group of taxonomists, whether amateur or professional, to develop and maintain our understanding of the state of biodiversity.

### A continuum of engagement

In the new context of citizen science and public engagement with science, we know very little about who participates in natural history and what motivates their continued volunteering, whether as an attractive but unpaid leisure activity or an accredited profession. A small number of authors have recently produced interesting work around motivations. For example, Dana Rotman et al. [26] argue that ‘volunteers participate in scientific activities out of interest, curiosity and commitment to conservation and related educational efforts’. Extending this further, Daniel Batson et al. [27] identify egotism, collectivism, altruism and principlism (upholding moral principles) as central underlying motivational factors for involvement with citizen science; whilst Jordan Raddick et al. [28] have studied motivations for involvement with GalaxyZoo, finding that contributing, learning, discovering, teaching others and perceiving the beauty and vastness of space were significant motivational factors for participants.

In this paper, we build upon these recent studies by drawing together recent work on the sociology of science and leisure studies in order to develop a continuum of engagement in citizen science for natural history, from the *previously*-*unengaged* participant who has never undertaken any citizen science work through the more *casually*-*engaged* participant who has been involved to a lesser degree in natural history or science in the past, to the strength and commitment of involvement frequently displayed by the *already*-*engaged* participant who in this instance may be described as a traditional amateur naturalist. We acknowledge the contribution of amateur naturalists to citizen science, and consider how participation can work to move people along this continuum in surprising and productive ways. We do so by examining the motivations behind citizen participation in the activities of the Open Air Laboratories (OPAL) programme, an England-wide, biodiversity monitoring and engagement project which began in 2007. Before we move on to our case study, we briefly outline the intellectual context for our research and findings.

### Citizen science and natural history

Although citizen science initiatives have exploded in number over the past 10–15 years, the practice has remained relatively under-represented in the peer-reviewed academic literature (cf. [9]: using Google Scholar, 2000–2009 produces 3420 results containing the phrase ‘citizen science’, whilst 2010–2014 produces 8750). Much of this work on citizen science has largely been written by professional scientists for professional scientists, in order to improve and argue for best practice in public involvement with projects, and allay fears surrounding data quality and reliability (see [5] for a review of citizen science environmental monitoring, cf. [29–32] for OPAL-related papers in this regard). A body of work is now emerging from within the social sciences on the more qualitative dimensions of what it means to participate in citizen science, shining a more critical light on how volunteering is understood not merely as an opportunity to increase data collection and manpower, but as a fundamental way in which people can work with and know the natural world [3, 33–36].

Recent work by sociologists of science and others has argued against the dichotomy of professional science’s interest in data versus humanistic concerns around motivation and participation [37, 38]. Indeed, this work and our paper seek to bridge the gaps between personal, embodied and emotional experiences of citizen science, wider political agendas, pressing environmental concerns and the demands for improved and increased scientific data and knowledge of the world. In order to make sense of the engagement continuum proposed above, which begins to account for the ways in which participants might remain or be transformed from previously-unengaged into casually- and perhaps already-engaged participants, we can usefully consider work around volunteering and leisure.

### Leisure studies

Leisure studies scholars identify volunteering as both unpaid work and attractive leisure. This offers a way of making sense of our continuum, specifically from the ‘serious leisure’ perspective, whereby leisure is categorised as either serious, casual or project-based. Leisure is understood by Robert Stebbins [39], as ranging from:

- *Serious leisure*: systematic pursuit of an amateur, hobbyist or volunteer activity sufficiently substantial, interesting and fulfilling for the participant to find a (leisure) career there, acquiring a combination of its specialist skills, knowledge and experience.
- *Casual leisure*: immediately, intrinsically rewarding, relatively short-lived pleasurable activity, requiring little or no special training to enjoy it.
- *Project*-*based leisure*: short-term, reasonably complicated, one-shot or occasional, though infrequent, creative undertaking carried out in free time or time free of disagreeable obligation.

We argue that citizen science activities, such as OPAL, form a major part of project-based leisure, whereby people are asked to participate in a scientific project that responds to either a pressing scientific question (such as the Soil and Earthworm Survey mapping worm populations) or urgent environmental challenge (such as the Tree Health Survey asking the public to report on tree health and harmful pests and diseases). However, our results reveal that OPAL is not only a form of project-based leisure; it also recruits individuals who may undertake forms of serious and casual leisure in the field of natural history and other associated topics. The empirical material here thus enables us to ask and understand: (i) how individuals encounter and experience the survey as a form of project-based leisure; (ii) what motivates them to take part and whether people volunteer as part of leisure, work or a sense of collective responsibility, and (iii) where volunteers feature on our continuum of engagement and in turn whether their participation facilitates their movement between the categories of previously-unengaged, casually-engaged and already-engaged. Furthermore, the inclusion of leisure studies perspectives ensures that the wide-ranging trials, tribulations, and commitments associated with citizen science are no longer overlooked in the desire to gather data for professional science projects.

In the race to herald citizen science as the panacea to many of science’s data problems, the figure of the amateur naturalist—as a serious leisure participant—cannot and should not be overlooked [40]. We begin by introducing OPAL, following this with a discussion of several instances of amateur involvement in OPAL. We then conclude the paper by arguing that this study offers a qualitative example of how citizen science can be meaningful to individuals beyond any public engagement and data collection exercise.

## Methods

As Davies et al. [41] outline in the first paper in this supplement, OPAL is one of the largest citizen science for natural history programmes ever attempted in England (cf. [1, 40, 42–44]). Unlike other biodiversity-focussed initiatives such as those of the BBC (Springwatch, Autumnwatch) and the RSPB’s Big Garden Birdwatch, OPAL differs in both its provision of materials asking people to follow an accessible yet formalised scientific methodology, and the diversity of fields covered. Further, OPAL’s team of regional community scientists act as key agents on the ground in the communication of science and engagement with the public. In this paper, we draw on qualitative research into OPAL activities, specifically focussing on those of OPAL North West (OPAL NW).

OPAL NW was one of nine OPAL regions in England operating during the programme’s first phase in 2007–2013. The NW team had the responsibility of distributing surveys and coordinating activities in the North West, as well as carrying out social research in the North West and West Midlands exploring how the thinking and behaviour of OPAL participants changed over time. The social research involved recorded focus groups, recorded in-person interviews in the two regions and telephone interviews with respondents from across the country, as wll as an online survey. All interactions took place around the principal ‘OPAL national citizen science’ surveys, and the link to the online survey was made available after people entered their data for these. The online survey was used to gain quick feedback from a maximum number of people close to the time of their doing a survey; it also allowed contacts to be gathered for later telephone interviews. Focus groups were used in addition to interviews to deepen understanding by drawing out reflections that might not have come out in a one-to-one interaction.

Five focus groups were held with 50 participants in total in the North West and West Midlands, and over 100 interviews were conducted. Six hundred online surveys were completed nationally, using mostly closed-response, agree-disagree questions with several free-text boxes where respondents could express briefly how they felt about activities. Fifty events or survey activities were also attended to enhance understanding and gain interview contacts. The research presented here is not intended to be representative of all OPAL participants; rather, it represents the views of a broad collection of participants in the North West and West Midlands that reveal the multiple ways in which people have engaged in the OPAL surveys.

The data was transcribed and then analysed in SPSS and NVivo as it became available using a Grounded Theory approach [45]; specifically, data-codes of significance are allowed to emerge from repeated readings of the transcriptions, rather than being imposed upon the data. In the following Results section, focus group data is marked as such and all named interviewees (using pseudonyms) are from either face-to-face or telephone interviews.

## Results

### The previously-unengaged participants

Feedback from OPAL participants reveals that the programme succeeded in engaging many people who previously had had no involvement with natural history. More than half of over 500 online survey respondents aged over 18 reported that OPAL was the first time they had participated in any such activity. The comments below from one online survey question illustrate some of the things people enjoyed about the activities and some reflections upon the motivations for their participation:

Q: What did you most enjoy about the OPAL survey activity?

‘Seeing my garden through different eyes’, ‘Learning about the natural world’, ‘I enjoyed seeing what was in the lake, being out in the fresh air, and doing the water sampling’, ‘Being able to identify what we found and feeling that by taking part we would be contributing to something useful’, ‘Participating was very interesting and I learned a few things. As a retired person it was nice to feel that I was part of a team of volunteers contributing to an important study’, ‘Learning something new and investigating familiar surroundings and seeing it in a different light’, ‘The chance to learn something new and to do something useful at the same time’.

These rich quotes relating to satisfaction with being outside, learning, observing new things and contributing data and time to a scientific project are representative of the general thrust of feedback and strongly supportive of Rotman et al., Batson et al. and Raddick et al.’s [26–28] findings. However the more in-depth data gathered from focus groups and interviews pointed at times to different elements in the overall picture. Interestingly, although three different methods of qualitative engagement were pursued in this research, no significant differences appeared between what people told us in focus groups, face-to-face and telephone interviews. The online survey did not elicit in-depth reflections, rather ‘vox-pop’ quotes, but this would be to be expected in such a more restricted interaction.

As outlined earlier, the social dimensions and motivations surrounding participation in citizen science remain still relatively unexplored. For this reason, the following section will consider one of the key challenges that emerged, namely a lack of time. For many OPAL participants, the experience of doing a survey was, as the quotes above suggest, so satisfying that they wanted to go on to do more. However as with all voluntary activity, it is exactly that: voluntary. Participants donate their time, energy and skill and are free to withdraw it at any time [46]. As the following examples attest, while the head and heart might be willing, often other pressures took priority such as family, leisure and work:

‘I mean, my life is incredibly busy at the moment. I think it’s the sort of thing I’d like to do when I’m retired’ [Bernice, 35-44]

‘I would like to do more but I don’t have the time to commit, so I think I would say at this point no.’ [Janet, 25-44]

‘I think my life is pretty full at the moment. I don’t feel that taking on anything else, I don’t think I would be able to do it justice’ [Patricia, 45-54]

Perceived lack of time is clearly a major factor influencing participation in projects where there is a commitment to being outdoors doing fieldwork. Even participants keenly aware of the environmental concerns underlying certain surveys often did not feel they could allow themselves to participate:

‘My day-job stops me doing more. If I had a job in environment and conservation I’d do more. I do as much as I can, I have very little free time. And my wife, although she works in gardening, planting trees and so on, she’s working all hours God sends as well, so I really don’t think we’ve got any time.’ [Dave, 35-44]

‘They’re all interesting. For me, if I was going to get involved in anything like that, it’s the time aspect … they’re all something I’d like to be involved in, but the practicalities of it, with the other commitments in my life.’ [Allotment-holders Focus Group]

These respondents struggle to justify contributing the spare time they *do* have to the OPAL surveys, juggling other pressures. However, the one-off, project-based nature of OPAL means the activities facilitate participation for time-pressed individuals.

### The casually-engaged participants

As mentioned, a key part of OPAL’s remit has been to engage the previously unengaged in natural history. A less expected but very significant outcome of OPAL’s work has been a further engagement of the casually-engaged amateur naturalist community. A key mechanism for enthusing the previously unengaged has been to draw on the success and passion of existing natural history societies and networks. In so doing, OPAL has come to the attention of many already casually-engaged individuals—developing, broadening and deepening their interests:

‘I’ve been involved with stuff to do with wildlife for a long time, but it’s been good, for really opening my eyes to what’s local to me … getting involved with OPAL encouraged me to want to brush up my knowledge … it’s enabled me to get back to doing something I loved doing a while ago, and I’ve kind of drifted – it certainly has got me more involved in things.’ [Cecilia, 35-44]

‘I think OPAL goes into more depth which is good, and feels more ‘sciencey’ [sic] – new word. It’s got me interested in going a bit further with researching, rather than just plopping about in a field or puddle, nice as these activities are. For me personally, as a failed science/biology student at school, it’s been a nice experience.’ [Diana, 35-44]

These interviewees highlight how OPAL has offered them significant experiences observing and monitoring nature, which has in turn given rise to increased confidence, renewed interest, refocused activity and validation. The power of citizen science with respect to empowerment cannot be underestimated. For many participants, increased confidence came from the purpose and satisfaction derived from contributing to a much larger dataset for a scientific project, valuing their records as ‘real science’:

‘I do care about the local environment, and I felt that I was going to be doing something useful … It’s something where I thought I could contribute to something bigger … which could create a database of, if lots of people got involved, the whole country.’ [Barbara, 35-44]

‘It’s given me a bit more confidence to do that sort of thing than I had before, because I feel I’m contributing … it’s a confidence booster really, because it helps me understand that I’m not as decrepit as I think I am sometimes.’ [Abigail, 65+]

Citizen science projects like OPAL clearly have a role to play in re-engaging those who have lost touch, or confidence in their abilities. The following respondent, for example, re-engaged with natural history through OPAL following the life event of having children:

‘I am very interested in the OPAL programme because of the opportunities it offers for education, re-acquainting myself with lost skills and giving a sense that one is making a difference by contributing to a wider research base.’ [Neil, 45-54]

The surveys further worked to engage those who had previously spent time outdoors for reasons other than natural history, key to arguments for the potential value in piggy-backing on the pre-existing interests and activities of the casually-engaged:

‘I was fascinated by [the OPAL Soil and Earthworm survey], because as an angler I knew there were lob worms and I knew there were brandlings, and the rest were just variations on a theme.’ [Paul, 55-64]

‘Before attending the OPAL activities and workshops, I went outside to enjoy the countryside, which usually involved following a ramblers trail … Post-OPAL interaction, I am now an active paid member of The Yorkshire Naturalists Union, Bumblebee Conservation Trust, Bat Conservation Trust … that’s only a selection of the activities!’ [Louis, 18-24]

It is clear from what has been said that participation in the OPAL surveys has empowered some previously-unengaged or casually-engaged individuals; in the next section we will highlight how OPAL has had comparable effects upon the already-engaged.

### The already-engaged participants

Participation in OPAL surveys has enabled the casually-engaged to broaden and deepen their interest and enthusiasm for natural history. For many already-engaged participants, the surveys offer a means of reframing their natural history activities for a different purpose and taking them out of their comfort zone to consider new areas they are unfamiliar with:

‘I would always have been doing natural history type things. I probably wouldn’t have done the pond-dipping, to be fair, without OPAL encouraging me – and having the nice little pack of stuff certainly encouraged me to go out and do the survey.’ [Martin, 55-64]

The ‘little pack of stuff’ is important to highlight further: as mentioned earlier, the OPAL survey packs, developed by the Field Studies Council, are regarded as relatively unique for incorporating a field notebook, field guide and other useful kit (such as a magnifying glass, compass, pencil and tape measure):

‘Well that’s what seduced me with OPAL really … the materials were so beautiful, I thought: ‘Oh, I’d really like to study this, so I get a better knowledge of what I’m looking at.’ [Brenda 55-64]

Even for some already-engaged participants, the OPAL surveys (literally or figuratively) expanded their toolkits:

‘I’ve always been interested in doing surveys … OPAL is just another string to my bow really, where I can seek advice or gain experience doing surveys. OPAL to me is another useful tool.’ [Martin, 35-44]

We have already highlighted how participation in citizen science can offer a way of renewing a pre-existing interest for the casually engaged. For the already engaged, OPAL surveys can go a step further:

‘It’s suddenly opening the box – it’s bottomless isn’t it? And I think that’s the beauty of it really, I’ll never learn as much as my enthusiasm wants me to learn … I’ve taken on too much now and I think my enthusiasm has outstripped my ability!’ [Adrian, 55-64].

Enthusiasm is infectious [47]. Participation in one OPAL survey begets increased participation in other surveys and so a widening of interests:

‘I’d most definitely like to know more – and organisations like OPAL have certainly helped me along that path … it’s an eye-opener, things I love learning … I’ve got nothing but admiration and praise for OPAL. I just wish we could reach all the people.’ [Steve, 55-64]

Participation is a social activity, whether between people and people, or between people and the natural world. For many respondents, OPAL worked as a means of opening up and building social networks:

‘What OPAL’s done for me is, whereas before I was a solitary naturalist, it’s introduced me to a lot more people who feel the same, who have got the same interests, so in that respect I think it’s absolutely brilliant.’ [Colin, 55-64]

‘[OPAL’s] helped me to see where I want to go with my career, it’s pushed me towards volunteering things … because of OPAL I met the nature person from the Council, and I’m doing a project with him now, [OPAL’s] kind of connected us.’ [April, 18-24]

Already-engaged individuals are likely to have developed some of the core skillsets required to undertake biodiversity monitoring activities and species identification. These participants will therefore be more likely to undertake the surveys with the required determination and patience to produce good quality results, as well as to recognise the importance of submitting these results.

Some of the respondents featured in this section form part of what Stebbins [39] describes as ‘serious leisure’ participants who are making a leisure career out of their interest, what might be termed a vocation. Their years of established experience in observation and recording and their associated networks remain invaluable to the continuing success of citizen science initiatives such as OPAL. This enthusiasm and experience can be key to encouraging previously-unengaged and more casually-engaged people to carry out surveys and increase their knowledge and abilities. OPAL has invested significantly in establishing good relationships with natural history societies, and these societies have in turn provided training and support for the more casually-engaged, as demonstrated by Leanne, who ran a small community group for her village:

‘I did the surveys for their educational aspects. They were great, professionally presented, everything in there, that made a big difference. But they were also good just for getting people involved, opening their eyes so they could see what was around them … With one group, we worked through the lichen survey and then they wanted to know more, so they got more materials and kept practising their ID skills. They have since done a lichen survey of the whole site!’ [Leanne, 45-54]

These already-engaged participants will bring years of established experience in observation and recording to the areas they now turn their eye to, as well as their networks of contacts who may also become interested. For new societies established alongside the OPAL programme such as the Earthworm Society of Great Britain, this will likely prove invaluable.

## Conclusions

OPAL’s aim of increasing participation in natural history is regarded by the environmental community, both amateur and professional, as sorely needed [26]. Long-term programmes of engagement such as OPAL are required in order to generate and retain significant attention and commitment to citizen science. Our research has demonstrated the potential for productive feedback to encourage advancement along our continuum between previously-unengaged, casually-engaged and already-engaged citizen science participants, producing opportunities for knowledge- and skill-sharing and thereby widening and deepening, as well as increasing, participation.

Our research echoes the academic literature on motivation identified earlier in this paper [26–28], revealing that there is no one-size-fits-all solution to increasing motivation for and participation in citizen science. However, our study identified the importance of projects like OPAL that combine public engagement and scientific endeavour in order to accommodate differing levels and rates of participation. Paying close attention to the new, relatively-new and established natural history participants identified here, OPAL and projects like it should continue to develop a range of approaches for different age-groups and demographics, designing and targeting their activities accordingly (see Davies et al. for examples of the approaches OPAL has engaged with thus far [41]).

Many of the issues highlighted in this paper are beyond the control of OPAL and its community scientists, survey-designers and project partners. OPAL is of course making strong contributions to encouraging shifts in thinking for people to find the time to engage in monitoring activities, creating the spaces and conditions for participation through project-based leisure that tackles important environmental questions [43], for example the health of the nation’s trees. However, as this paper has argued, interest, motivation and a sense of collective responsibility can never be guaranteed (Ibid.). The full potential of citizen science is yet to be realised, however the example of OPAL reveals the power of participation in citizen science to move volunteers between the categories of previously-unengaged, casually-engaged and already-engaged. The success of this continuum of engagement should not be underestimated as the rewards for participation range from a personal sense of achievement to the contribution to ‘real’ scientific research.

## Abbreviations

ID: identification
OPAL: open Air Laboratories
RSPB: Royal Society for the Protection of Birds
SPSS: statistical package for the social sciences
